# Maternal Smoking during Pregnancy and Necrotizing Enterocolitis-associated Infant Mortality in Preterm Babies

**DOI:** 10.1038/srep45784

**Published:** 2017-03-31

**Authors:** Guodong Ding, Jing Yu, Yan Chen, Angela Vinturache, Yu Pang, Jun Zhang

**Affiliations:** 1 MOE and Shanghai Key Laboratory of Children’s Environmental Health, Xinhua Hospital, Shanghai Jiao Tong University School of Medicine, Shanghai 200092, China; 2 Department of Pediatrics, Shanghai East Hospital, Tongji University School of Medicine, Shanghai 200120, China; 3 Department of Endocrinology and Metabolism, Shanghai Jiao Tong University Affiliated Sixth People’s Hospital, Shanghai 200233, China; 4 Department of Obstetrics & Gynaecology, John Radcliffe Hospital, Oxford University Hospital Trust, Headley Way, Oxford, OX3 9DU, UK; 5 China Novartis Institutes for BioMedical Research Co., Ltd, Shanghai 201203, China

## Abstract

Few studies have examined the possible pregnancy-related risk factors for necrotizing enterocolitis (NEC)-associated deaths during infancy. Infant death due to NEC in preterm babies was identified from the US Linked Livebirth and Infant Death records between 2000 and 2004. The average number of cigarettes per day reported by the mothers who were smoking during pregnancy was classified in three categories: non-smoking, light smoking (<10 cigarettes/day) and heavy smoking (≥10 cigarettes/day). Logistic regression analyses examined the association between prenatal smoking and NEC-associated infant mortality rates with adjustment for potential confounders. Compared with non-smoking mothers, light and heavy smoking mothers have a higher risk of NEC-associated infant mortality [light smoking: adjusted odds ratio (aOR) = 1.21, 95% confidence interval (CI), 1.03–1.43; heavy smoking: aOR = 1.30, 95% CI, 1.12–1.52], respectively. Moreover, the association was stronger among white race (light smoking: aOR = 1.69, 95% CI, 1.34–2.13; heavy smoking: aOR = 1.44, 95% CI, 1.18–1.75) and female babies (light smoking: aOR = 1.31, 95% CI, 1.02–1.69; heavy smoking: aOR = 1.62, 95% CI, 1.29–2.02). Maternal smoking during pregnancy is associated with increased risks of infant mortality due to NEC in preterm babies, especially in white race and female babies.

Necrotizing enterocolitis (NEC) is the most common gastrointestinal emergency and an important cause of morbidity and mortality in neonates^1^. In spite of remarkable advances in the diagnosis and treatment of NEC, the mortality rate due to NEC of about 15–25% did not change appreciably in the last 30 years^2^. NEC has proven one of the most difficult to prevent diseases in neonates and has become a priority for research^3^.

The causative factors of NEC remain elusive^4^. Hypoxic-ischaemic injury, feeding with formula milk, and colonisation by pathological bacteria have been commonly identified as contributing factors^4^. Prematurity and low birthweight increase the risk of NEC due to developmental immaturity of key functions such as intestinal function, circulatory regulation, and immune defense in these infants. The rates of NEC-associated mortality are even higher (15.9 per 10,000) in low birthweight and premature babies^5^. Several well-designed studies have identified a range of prenatal events and maternal risk factors such as smoking, age, high BMI, gestational diabetes, and hypertension that predispose infants to develop NEC^6^,^7^,^8^. However, less is known about the role of these factors in NEC-associated mortality in the premature infants.

It is well established that maternal smoking during pregnancy is frequently associated with adverse pregnancy outcomes including, but not limited to, preterm birth, fetal growth restriction, and various placental conditions, resulting in increased risks of infant morbidity and death^9^,^10^,^11^,^12^. Nicotine can readily pass through the placenta and transfer to the fetus, exposing the fetus to higher levels of nicotine than in the mother^13^, rendering the fetus susceptible to direct poisoning. In addition, prenatal nicotine exposure impairs placental development by changing the balance between cytotrophoblast proliferation and differentiation and by reducing the blood flow, creating a pathologically hypoxic environment^14^. Despite the risks, a considerable number of women continue to smoke when they become pregnant. As much as one-fourth to two-thirds women who smoke prior to pregnancy will continue to smoke while pregnant^15^,^16^.

To date, only few epidemiological studies investigated the possible associations between maternal smoking during pregnancy and NEC with inconsistent results, some reporting a negative relationship^6^, while others showing no association^17^.

We hypothesized that maternal smoking during pregnancy increases the risk of death in NEC-affected premature babies. Thus, this population-based cohort study was designed to examine the potential relationship between maternal smoking throughout pregnancy and NEC-associated infant death in preterm babies.

## Methods

In this study, we used the data from the US Linked Livebirth and Infant Death files from 2000 to 2004, which included all births in the USA and linked infant (<1 year of age) deaths, recorded by death certificates^18^. The dataset was published by the National Center for Health Statistics (NCHS), US Centers for Disease Control and Prevention (CDC). A detailed description of this data is provided elsewhere^18^. The available information in this data repository included demographic characteristics of mothers, obstetric history, maternal smoking, birth outcomes, infant death, and underlying cause of death.

Maternal smoking identified on the birth certificate included a “yes” or “no” response and the average number of cigarettes smoked per day during pregnancy. We coded maternal smoking during pregnancy as a trichotomous variable: (1) nonsmokers, (2) light smokers, women who reported smoking 1 to 10 cigarettes per day during pregnancy, and (3) heavy smokers, women who reported smoking more than 10 cigarettes per day during pregnancy. Maternal smoking was not reported in California, Indiana, South Dakota, and New York State (except New York City) during the study period. These subjects were excluded from the analysis.

Women were classified in three groups based on maternal age at delivery: 19 years or less, 20–34 years, and 35 years or older. Maternal race/ethnicity was classified as non-Hispanic white, non-Hispanic black, Hispanic, and Asian. Maternal educational attainment was collapsed into four categories: less than high school (<12 years), completed high school ( = 12 years), college (13–16 years), and graduate school (≥17 years). Marital status was recorded as a dichotomos variable (married/unmarried). Infant birthweight was expressed in grams and infant gender was recorded as male/female. Gestational age at delivery was estimated based on the date of the last menstrual period (LMP); if the LMP was unreliable or if there was a substantial discordance between the clinical estimate of gestational age and LMP (>2 weeks), the first clinical estimate of gestational age was used^19^. Based on the month when prenatal care services were first accessed, women were classified into four groups: received prenatal care from first trimester of pregnancy (months 1–3), from the second trimester (months 4–6), from the third trimester (months 7–9), and no prenatal care.

NEC-associated infant deaths (<1 year of age) were defined as deaths for which International Classification of Diseases ICD-10 code P77 was recorded on the death certificate^20^. NEC-associated infant deaths were further divided into three categories: early neonatal death (0–6 days), late neonatal death (7–27 days), and postneonatal death (28–364 days).

**Figure 1:**
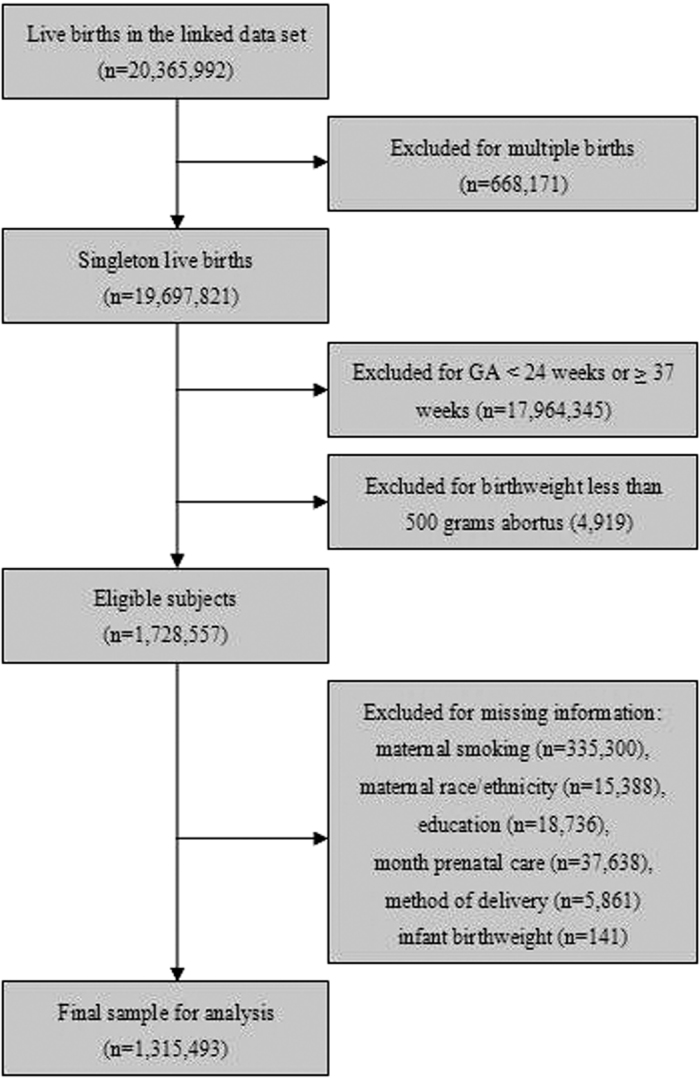
Flow chart of inclusion and exclusion criteria of the subjects, Birth and Infant Death Cohort, 2000–2004.

We restricted our analysis to singleton live births at gestational ages of 24–36 weeks and with birthweights of 500 g or more. There were 20,365,992 live births in the linked 2000–2004 birth and infant death data set (Fig. 1). We excluded 668,171 multiple gestations, 17,964,345 newborns delivered before 24 or after 36 weeks of gestation, and 4,919 births of less than 500 g. Subjects with missing information on important variables including maternal smoking (335,300), maternal race/ethnicity (15,388), education (18,736), month prenatal care (37,638), method of delivery (5,861), and infant birthweight (141) were further excluded, leaving 1,315,493 subjects for the final analysis.

NEC infant mortality rates, calculated as the number of deaths per 10,000 live births, were determined overall and by several variables such as birthweight, gestational age, infant sex, and maternal race/ethnicity. Unconditional logistic regression models were constructed to examine the association between maternal smoking during pregnancy and risk of NEC-associated infant mortality. Odds ratios (ORs) and the corresponding 95% confidence intervals (CIs) were computed after adjusting for potential confounders. The confounders were chosen for inclusion in the adjusted models based on information from previous literature: maternal race/ethnicity, age, education, marital status, month that prenatal care started, infant sex, gestational age, and birth year^9^,^10^,^11^,^12^. Infants born to non-smoking mothers were the reference group. Cox proportional hazard model was used to estimate risk ratio of infant mortality among the nonsmokers, light smokers, and heavy smokers, adjusting for the above confounders. All analyses were performed with Statistical Analysis System (version 9.2, SAS Institute, Cary, N.C.).

Since these files contain anonymized public data, an ethics approval was not required by an Institutional Review Board.

## Results

Table 1 presents the characteristics of preterm pregnancies (24–36 gestational weeks) resulted in live births and NEC-related infant mortality in the USA from 2000 to 2004. From a total of 1,315,493 eligible singletons, live preterm babies, 172,096 (13.1%) were exposed to prenatal smoking. Within the first year of life, 1,122 NEC-associated deaths were recorded leading to a mortality rate of 8.5 per 10,000 live births due to this cause. The majority (82.7%) of infant deaths due to NEC occurred in the late neonatal period, 7 to 27 days after birth.

Maternal race/ethnicity comprised of non-Hispanic White (55.9%), non-Hispanic Black (23.4%), Hispanic (17.1%) and Asian (3.7%). Nearly three-quarters (72.3%) of the women were between 20 and 34 years old, and two-thirds (59.2%) were married. The majority of women have attained education at the level of high school or higher (76.5%), and attended prenatal care from the first trimester of pregnancy (80.8%). Approximately 83% of preterm deliveries were late preterm (33 to 36 weeks), little over half (53.9%) of the preterm babies were male.

Supplementary Table 1 compares characteristics between smokers and non-smokers. Several characteristics including maternal age, race/ethnicity, education, marital status, prenatal care, infant sex, gestational age, and birthweight were significantly different between the two groups. Compared with non-smokers, smokers were less likely to attain high educational level and attend prenatal care, and more likely to be white, single, and young childbearing age.

The association between maternal smoking during pregnancy and the risk of NEC-associated infant mortality is presented in Table 2. Compared with non-smoking, light and heavy smoking was associated with increased risks of NEC infant mortality [light smoking: adjusted OR (aOR) = 1.21, 95% CI, 1.03–1.43; heavy smoking: aOR = 1.30, 95% CI, 1.12–1.52], respectively. Further analyses of the relationship between maternal smoking and the NEC-associated infant mortality were stratified by birthweight, gestational age, infant sex, and maternal race/ethnicity. We found that the association was stronger among white race (light smoking: aOR = 1.69, 95% CI, 1.34–2.13; heavy smoking: aOR = 1.44, 95% CI, 1.18–1.75) and female babies (light smoking: aOR = 1.31, 95% CI, 1.02–1.69; heavy smoking: aOR = 1.62, 95% CI, 1.29–2.02).

Using the Cox proportional hazard models, we analyzed the survival curves among the nonsmokers, light smokers, and heavy smokers stratified by (white) race and (female) gender of the babies. The three survival curves were similar, showing a slight difference of NEC-associated infant mortality among the three maternal groups (Supplementary Figures 1 and 2).

## Discussion

In the present study, we examined the potential relationship of maternal smoking during pregnancy with NEC-associated infant mortality in preterm babies. Our results suggest that increased risk of infant mortality due to NEC is associated with smoking during pregnancy, both light and heavy prenatal smoking. This association appears to be stronger among infants of female gender and white race. The Cox proportional hazard models showed a slight difference of NEC-associated infant mortality among the three maternal groups.

A previous case-control study from the US identified maternal smoking during pregnancy as the only risk factor that was associated with the development of NEC in infants^6^. In contrast, a recent Canadian retrospective cohort study, examining the adverse impacts of maternal cigarette smoking on preterm infants, found no association between maternal smoking and NEC-associated mortality^17^. Our results support the relationship of prenatal smoking with NEC-associated mortality in preterm infants. Although studies on maternal smoking during pregnancy and infant mortality are not new, the mechanism through which tobacco causes death during infancy is still poorly understood. The proposed biological mechanisms include fetal hypoxia due to increased levels of carboxyhemoglobin and resulting decreased levels of blood oxygen, nicotine induced placental vasoconstriction, and placental vascular diseases leading to short- and long-term adverse effects on their offspring health^21^.

The effect of maternal smoking during pregnancy on the developing placenta has long been of clinical interest. Nicotine exposure impairs the initial stage of placental vascularization, the invasion of maternal blood vessels into the cytotrophoblasts^6^,^14^. Nicotine from the mother can also readily cross the placenta and transfer to the fetus, rendering the fetus susceptible to direct poisoning^13^. Maternal cigarette smoking has been related with lower gestational age at birth, decreased birth weight, and shorter crown-heel length in many studies^22^,^23^. Despite the aforementioned studies linking smoking to abnormal vascular development, few studies actually show a causality for the mechanism for this association.

We further found that the association between maternal smoking during pregnancy and NEC-associated infant mortality was stronger among white race and female babies. Although it is not clear what biological mechanisms could explain these findings, gender- and racial-specific effects of prenatal smoking on infant deaths due to NEC may exist. In previous animal studies, nicotine was showed to have sex-dependent effects on offspring health. Perters and Tang (1982) found that prenatal nicotine treatment of adult female rats reduced both the number of male born and male birth weight, whereas female offsprings tended to be unaffected^24^. Subsequently, several human studies suggested that the adverse effect of prenatal smoking on offspring growth and development was more pronounced among males than females^25^,^26^,^27^. However, a recent large study (n = 1,815,318), using the 1995 - 1997 German birth statistics, revealed that the negative effect of maternal smoking during pregnancy on the birth weight and risk of small-for-gestational-age (SGA) was significantly greater in newborn female than in newborn male^28^. Thus, our findings are consistent with the latter study.

Regarding the racial and ethnic differences in the association between maternal smoking during pregnancy and offspring health, there have been inconsistent findings. While some studies reported that the negative effect was greater for black children than for white children^29^,^30^, others reported stronger associations among white than black children^31^,^32^. In the present study, the association between maternal smoking and NEC-associated infant mortality was stronger among white babies as compared with the babies from other ethnic/racial groups. The following two factors may explain the differences found across race/ethnicity categories. Firstly, different genetic polymorphisms affecting nicotine metabolism probably exists among different racial/ethnic groups^33^. Variations in enzyme activity could alter the toxic effects on the offspring. Secondly, dose of nicotine per cigarette may vary, for instance due to racial/ethnic group preferences for certain types of cigarettes^34^. Thus, the same quantity of cigarettes may not mean the same dose of toxins across various groups.

We also observed that 82.7% of infant deaths occurred in the late neonatal period (i.e., in infants who survived early causes of mortality). This pattern is consistent with the expected timing of NEC onset reported in the literature^5^. Despite the decline in the overall infant mortality observed during the last several decades^5^, improvements in survival rates of very early preterm/low birth weight babies from early causes of death, may increase the rates of NEC-associated mortality in these immature infants, and explain the persistent high mortality rate (8.5 per 10,000 live preterm singleton births) reported here and elsewhere^5^. Intervention strategies to decrease the incidence of prematurity may have a potential greater impact on reducing the number of NEC-associated morbidity and mortality.

The large population-based database linking birth and infant death data is a major strength of our study. However, some limitations are worth mentioning. Firstly, smoking on the birth certificates was self-reported, which may have introduced some reporting bias, and potentially underestimated the percentage of smokers in pregnancy, and, therefore, likely affected the reliability of smoking prevalence. With the highly advertised adverse perinatal effects of smoking in pregnancy and the mounting social and medical pressure on women to quit smoking during pregnancy, the accuracy of the information on smoking during pregnancy may be questionable^35^. Secondly, uncontrolled genetic and environmental confounding effects may exist. For example, human milk is the preferred diet for preterm babies as it protects against NEC, but we are unable to control for the type of feeding or breastfeeding rate. Evidence from observational and trial data suggests that human milk diet reduces the incidence and severity of NEC^5^,^36^. A dose-related association was reported between human milk feeding and a reduction of risk of NEC or death after the first 2 weeks of life among extremely low birth weight infants^37^. In our study, we did not control for the type of feeding, as no data regarding feeding and nutrition was available; thus, we acknowledge here this limitation of our study. Smoking may be a proxy for other exposures such as alcohol and stress, for which there are no reliable measures. Finally, a diagnosis of NEC could include Bell’s stage I, which encompassed a significant proportion of non-NEC entities. However, by focusing on NEC mortality, this criticism is largely obviated. NEC as a cause of mortality is a clearly defined entity. While the coincidence with other conditions may have occurred (i.e., sepsis first, NEC second), it would likely be infrequent.

In summary, our study demonstrates that maternal smoking during pregnancy is associated with NEC-specific infant mortality in premature babies. These findings may have implications in basic and epidemiologic research on the mechanisms that underlie NEC-associated development and death.

## Additional Information

**How to cite this article**: Ding, G. *et al*. Maternal Smoking during Pregnancy and Necrotizing Enterocolitis-associated Infant Mortality in Preterm Babies. *Sci. Rep.* **7**, 45784; doi: 10.1038/srep45784 (2017).

**Publisher's note:** Springer Nature remains neutral with regard to jurisdictional claims in published maps and institutional affiliations.

## Supplementary Material

Supplementary Information

## Figures and Tables

**Table 1:** Characteristics of the preterm pregnancies resulted in live births and NEC-related infant mortality, Birth and Infant Death Cohort, 2000–2004.

Characteristic	Live birth (n = 1,315,493)	NEC infant death (n = 1,122)	Early neonatal death 0–6 days (n = 83)	Late neonatal death 7–27 days (n = 928)	Postneonatal death 28–364 days (n = 111)
Maternal smoking					
Non-smokers	86.9%	83.5%	84.3%	83.4%	83.8%
Light smokers	5.3%	7.3%	7.2%	7.4%	6.3%
Heavy smokers	7.8%	9.2%	8.4%	9.2%	9.9%
Maternal age, years					
≤ 19	13.3%	19.4%	16.9%	19.2%	23.4%
20–34	72.3%	68.8%	67.5%	68.9%	69.4%
≥35	14.4%	11.8%	15.7%	12.0%	7.2%
Maternal race/ethnicity					
Non-Hispanic White	55.9%	34.7%	31.3%	34.4%	39.6%
Hispanic	17.1%	15.9%	20.5%	15.5%	15.3%
Non-Hispanic Black	23.4%	46.0%	43.4%	46.4%	44.1%
Asian	3.7%	3.5%	4.8%	3.7%	0.9%
Maternal education, years					
<12	23.5%	32.5%	33.7%	32.7%	30.6%
=12	33.3%	37.0%	44.6%	35.7%	42.3%
13–16	34.9%	26.3%	19.3%	27.3%	23.4%
≥17	8.2%	4.2%	2.4%	4.4%	3.6%
Maternal marital status					
Married	59.2%	40.8%	42.2%	41.4%	35.1%
Unmarried	40.8%	59.2%	57.8%	58.6%	64.9%
Month that prenatal care started					
1st Trimester	80.8%	77.8%	73.5%	78.8%	73.0%
2nd Trimester	13.6%	15.2%	21.7%	14.4%	17.1%
3rd Trimester	2.8%	1.4%	2.4%	1.4%	0.9%
No prenatal care	2.9%	5.5%	2.4%	5.4%	9.0%
Gestational age at delivery, weeks					
24–26	3.0%	41.3%	14.5%	42.1%	54.1%
27–29	4.4%	32.9%	15.7%	33.9%	36.9%
30–32	9.4%	16.4%	27.7%	16.4%	8.1%
33–36	83.1%	9.4%	42.2%	7.5%	0.9%
Infant sex					
Male	53.9%	58.1%	61.4%	58.3%	54.1%
Female	46.1%	41.9%	38.6%	41.7%	45.9%

**Table 2:** Association of maternal light and heavy smoking and NEC infant mortality in preterm babies, Birth and Infant Death Cohort, 2000–2004.

	Smoking						Non-smoking			Adjusted OR	
	Light smoking (<10 cigs/day)			Heavy smoking (≥10 cigs/day)						<10 cigs/day	≥10 cigs/day
	Death case	Live birth	Mortality rate (per 10,000)	Death case	Live birth	Mortality (per 10,000)	Death case	Live birth	Mortality (per 10,000)	OR (95% Cl)	OR (95% Cl)
Total^a^	82	69,589	11.8	103	102,507	10.0	937	1,143,397	8.2	**1.21 (1.03, 1.43)**	**1.31 (1.12, 1.52)**
Birthweight (g)^b^											
<750	16	1,404	114.0	20	1,697	117.9	263	20,858	126.1	0.83 (0.58, 1.20)	0.92 (0.66, 1.30)
750–999	24	2,028	118.3	34	2,728	124.6	263	29,002	90.7	1.29 (0.95, 1.76)	**1.58 (1.20, 2.09)**
1000–1499	34	4,889	69.5	33	6,977	47.3	265	66,825	39.7	**1.64 (1.26, 2.13)**	**1.37 (1.04, 1.81)**
1500–1999	5	9,178	5.4	11	13,827	8.0	95	120,185	7.9	0.64 (0.33, 1.22)	1.11 (0.69, 1.78)
2000–2499	3	19,562	1.5	3	30,331	1.0	36	257,715	1.4	1.27 (0.54, 2.98)	1.09 (0.45, 2.64)
≥2500	0	32,528	—	2	46,947	0.4	15	648,812	0.2	—	1.66 (0.54, 5.09)
Gestational age, wk^c^											
24—26	26	2,329	111.6	36	3,005	119.8	401	34,248	117.1	0.92 (0.69, 1.22)	1.12 (0.86, 1.45)
27—29	39	3,479	112.1	32	4,752	67.3	298	50,134	59.4	**1.75 (1.37, 2.24)**	1.19 (0.90, 1.56)
30—32	13	7,366	17.6	29	10,480	27.7	142	106,208	13.4	1.23 (0.82, 1.86)	**2.39 (1.75, 3.26)**
33—36	4	56,415	0.7	6	84,270	0.7	96	952,807	1.0	0.60 (0.29, 1.23)	0.80 (0.43, 1.47)
Infant sex^d^											
Male	46	37,151	12.4	52	54,834	9.5	554	616,626	9.0	1.15 (0.92, 1.43)	1.10 (0.88, 1.36)
Female	36	32,438	11.1	51	47,673	10.7	383	526,771	7.3	**1.31 (1.02, 1.69)**	**1.62 (1.29, 2.02)**
Maternal race/ethnicity^e^											
White	45	43,732	10.3	73	85,745	8.5	271	605,295	4.5	**1.69 (1.34, 2.13)**	**1.44 (1.18, 1.75)**
Hispanic	2	5,306	3.8	7	3,227	21.7	169	216,019	7.8	0.41 (0.15, 1.12)	**2.42 (1.40, 4.20)**
Black	34	19,496	17.4	22	12,777	17.2	460	275,061	16.7	1.02 (0.79, 1.31)	1.02 (0.75, 1.40)
Asian	1	1,055	9.5	1	758	13.2	37	47,022	7.9	0.97 (0.23, 4.17)	1.15 (0.25, 5.33)

^a^OR adjusted for maternal race/ethnicity, age, education, marital status; prenatal care began, gestational age, infant sex, and birth year.

^b^OR adjusted for maternal race/ethnicity, age, education, marital status; prenatal care began, gestational age, infant sex, and birth year.

^c^OR adjusted for maternal race/ethnicity, age, education, marital status; prenatal care began, infant sex, and birth year.

^d^OR adjusted for maternal race/ethnicity, age, education, marital status; prenatal care began, gestational age, and birth year.

^e^OR adjusted for maternal age, education, marital status; prenatal care began, gestational age, infant sex, and birth year.
