# Supplementary material for: Maternal Smoking during Pregnancy and Necrotizing Enterocolitis-associated Infant Mortality in Preterm Babies
Source: Sci Rep. 2017 Mar 31;7:45784. doi: 10.1038/srep45784 (PMC5374458; doi:10.1038/srep45784)
Supplement: Supplementary Information [file srep45784-s1.pdf]

Maternal Smoking during Pregnancy and Necrotizing Enterocolitis-associated Infant Mortality in Preterm Babies

Guodong Ding[1,2 #], Jing Yu[3#], Yan Chen[1#], Angela Vinturache[4], Yu Pang[5], Jun Zhang[1*]

[1]MOE and Shanghai Key Laboratory of Children's Environmental Health, Xinhua Hospital, Shanghai Jiao Tong University School of Medicine, Shanghai 200092, China

[2]Department of Pediatrics, Shanghai East Hospital, Tongji University School of Medicine, Shanghai 200120, China

[3]Department of Endocrinology and Metabolism, Shanghai Jiao Tong University Affiliated Sixth People's Hospital, Shanghai 200233, China

[4]Department of Obstetrics & Gynaecology, John Radcliffe Hospital, Oxford University Hospital Trust, Headley Way, Oxford, OX3 9DU, UK

[5]China Novartis Institutes for BioMedical Research Co., Ltd, Shanghai 201203, China

---

[#]Guodong Ding, Jing Yu, and Yan Chen contributed equally to this work.

*Address correspondence to: Dr. Jun Zhang, MOE and Shanghai Key Laboratory of Children's Environmental Health, Xinhua Hospital, Shanghai Jiao Tong University School of Medicine, 1665 Kongjiang Road, 200092 Shanghai, China. Tel.: +86 21 25078871; fax: +86 21 25078875.

E-mail addresses:

dingguodong204296@126.com (Guodong Ding), yujing_tj@163.com (Jing Yu), chenyan783563@163.com (Yan Chen), angela.vinturache@doctors.org.uk (Angela Vinturache), pangyu911@126.com (Yu Pang), junjimzhang@sina.com (Jun Zhang).

Supplementary Table 1. Comparison of the characteristics of non-smoking and smoking mothers with singleton, preterm pregnancies, Birth and Infant Death Cohort, 2000–2004*

Characteristic	Non-smoking mothers (n= 1,143,397)	Smoking mothers (n= 172,096)
**Maternal age, y**		
≤ 19	148,967 (13.0%)	26,384(15.3%)
20–34	826,412 (72.3%)	124,287 (72.2%)
≥ 35	168,018 (14.7%)	21,425 (12.5)
**Maternal race/ethnicity**		
Non-Hispanic White	605,295 (52.9%)	129,477 (75.2%)
Hispanic	216,019 (18.9%)	8,533 (5.0%)
Non-Hispanic Black	275,061 (24.1%)	32,273 (18.8%)
Asian	47,022 (4.1%)	1,813 (1.1%)
**Maternal education, y**		
< 12 (Less than high school)	242,631 (21.2%)	66,961 (38.9%)
= 12 (High school)	366,560 (32.1%)	72,054 (41.9%)
13–16 (College)	427,653 (37.4%)	31,522 (18.3%)
≥ 17 (Graduate school)	106,553 (9.3%)	1,559 (0.9%)
**Maternal marital status**		
Married	712,151 (62.3%)	67,235 (39.1%)
Unmarried	431,246 (37.7%)	104,861 (60.9%)
**Month that prenatal care started**		
1st Trimester	940,526 (82.3%)	121,886 (70.8%)
2nd Trimester	146,126 (12.8%)	32,354 (18.8%)
3rd Trimester	29,642 (2.6%)	7,357 (4.3%)
No prenatal care	27,103 (2.4%)	10,499 (6.1%)
**Gestational period ,wk**		
24—26	34,248 (3.0%)	5,334 (3.1%)
27—29	50,134 (4.4%)	8,231 (4.8%)
30—32	106,208 (9.3%)	17,846 (10.4%)
33—36	952,807 (83.3%)	140,685 (81.8%)
**Infant sex**		
Male	616,626 (53.9%)	91,985 (53.5%)
Female	526,771 (46.1%)	80,111 (46.6%)
**Birthweight, g**		
<750	20,858 (1.8%)	3,101 (1.8%)
750–999	29,002 (2.5%)	4,756 (2.8%)
1000–1499	66,825 (5.8%)	11,866 (6.9%)
1500–1999	120,185 (10.5%)	23,005 (13.4%)
2000–2499	257,715 (22.5%)	49,893 (29.0%)
≥ 2500	648,812 (56.7%)	79,475 (46.2%)

*All differences between smoking and non-smoking mothers were statistically significant at $p<0.001$.

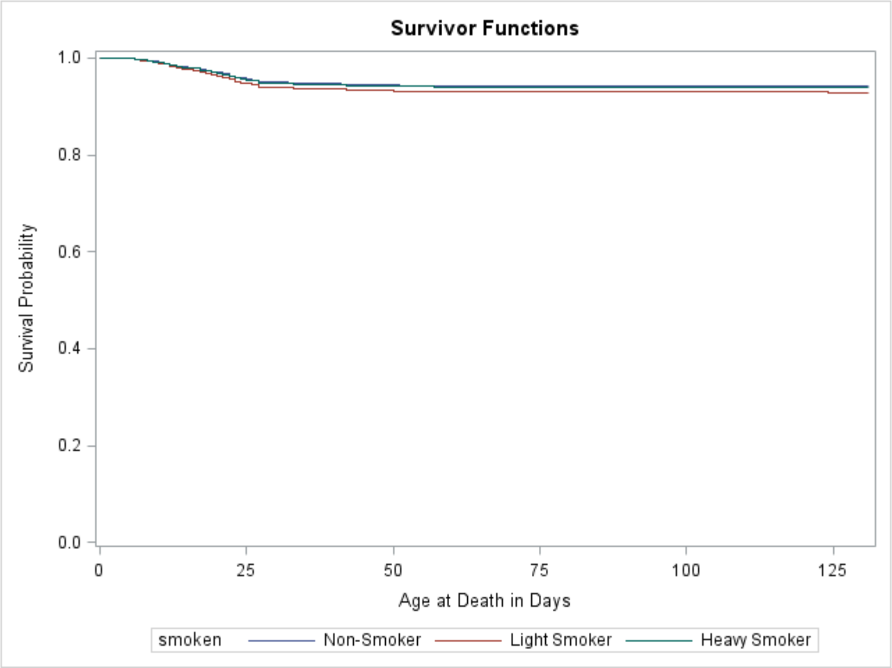

Supplementary figure 1. Survival curves among the nonsmokers, light smokers, and heavy smokers in white race

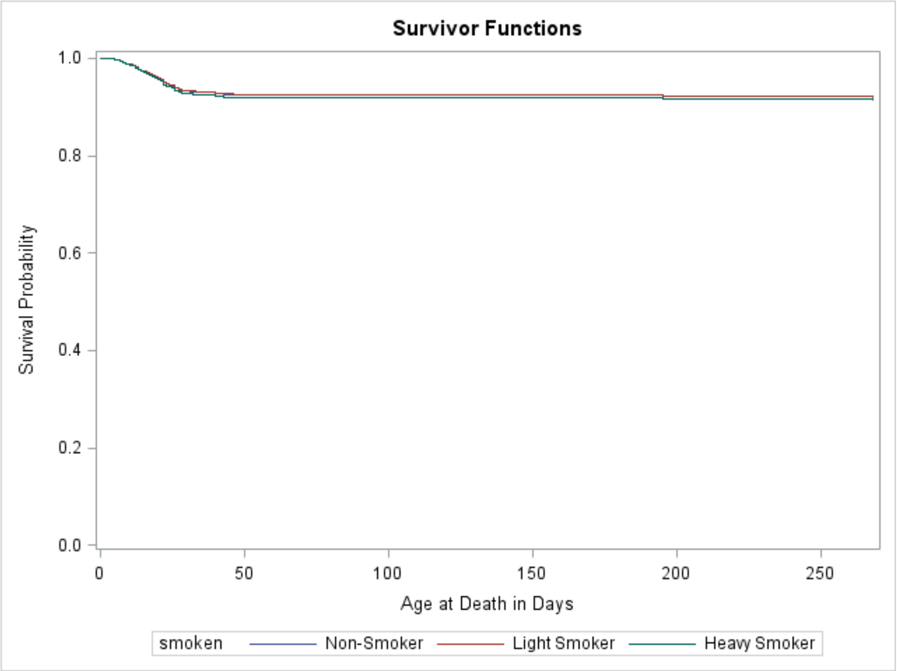

Supplementary figure 2. Survival curves among the nonsmokers, light smokers, and heavy smokers in female gender
